# Supplementary material for: Are carbon emissions trading and green financial instruments synergistic? -Comprehensive quantitative research based on content analysis
Source: PLoS One. 2024 Mar 7;19(3):e0298601. doi: 10.1371/journal.pone.0298601 (PMC10919601; doi:10.1371/journal.pone.0298601)
Supplement: S1 Data — (ZIP) [file pone.0298601.s001.zip › Data/地级市Z_F工作报告.zip!/╣π╢1⁄2/║╙╘┤╩╨/└┤╫╘╣1⁄2╓┌║┼í╛┬φ┐╦╩2╛▌═°í┐_2019.docx]

各位代表：

现在，我代表市人民政府向大会作政府工作报告，请予审议，并请市政协各位委员和其他列席人员提出意见。

一、2018年工作回顾

过去一年，我们紧密团结在以习近平同志为核心的党中央周围，坚持把贯彻落实习近平新时代中国特色社会主义思想和党的十九大精神作为全年工作主线，深入贯彻习近平总书记重要讲话精神，坚持稳中求进工作总基调，坚持新发展理念，全面落实省委、省政府决策部署，在市委的坚强领导下，在市人大及其常委会和市政协的监督支持下，全力克服各种不利因素，在艰难中拼力前行，在发展中谋篇布局，推动经济社会发展迈上新台阶。

（一）狠抓发展第一要务，生产总值突破千亿。

积极应对经济大环境的新情况新变化，始终坚持目标导向和问题导向，经过努力拼搏，实现GDP超千亿元目标。

经济发展态势向好。预计全市实现生产总值1002亿元，同比增长6.2%。完成固定资产投资677亿元，增长10%。社会消费品零售总额629.6亿元，增长8.8%。进出口总额270.6亿元，增长3.9%。一般公共预算收入76.95亿元，增长8.1%。民营经济实现增加值650亿元，增长7.5%，占GDP比重达65%。居民消费价格涨幅2%。居民人均可支配收入19336元，增长9.1%；其中城镇居民人均可支配收入25492元，增长7.2%。

经济运行质量提升。经济结构进一步优化，第三产业占比提高1.7个百分点。高技术制造业增加值和先进制造业增加值占规上工业增加值比重分别达38.1%和52.3%。主营业务收入超亿元、超10亿元的企业分别达220家、20家，纳税额超千万、超亿元的企业分别达112家、9家，在资本市场挂牌企业113家。

现代服务业加快发展。现代服务业增加值占服务业比重达48.5%，比去年提高1.5个百分点。编制完成全市全域旅游发展规划，智慧旅游平台上线运营，成功举办第十三届客家文化旅游节。全年接待入市游客3587万人次，增长12.1%；实现旅游总收入316.7亿元，增长16.1%。新增限额以上贸易业单位118家。保税物流中心、无水港等平台加快建设，跨境电商、保税物流等外贸新业态加快发展。

（二）狠抓园区和项目建设，发展基础不断夯实。

以园区为主战场，以项目为主抓手，着力掀起大抓工业、大抓项目、大抓招商的热潮。

工业发展势头趋好。实现规上工业增加值302亿元，增长7.8%；完成工业固定资产投资201亿元，增长15%；工业技改投资增长10%；增速均在全省排名前列。新增规上工业企业70家。主导产业不断壮大，电子信息产业年产值500亿元。

园区提质增效明显。新增连平（南山）省级产业园，江东新区产业园纳入深圳河源产业共建范围。全市8个省级产业园实现规上工业增加值超250亿元，增长8.5%；实现税收入库24亿元，增长11.3%；完成园区设施投入25亿元，实现“三通一平”8平方公里；园区新签约项目107个、新开工93个、新投产50个。5个省级产业园获省考评优秀。深圳对口帮扶完成产业转移项目54个，已落地建设的投资超亿元工业项目48个，完成率240%，全省排名第一。市高新区和连平县分别与深圳南山共建市场化“飞地经济”，由深圳国企承建、招商和运营。

招商引资成果喜人。出台招商引资优惠政策“黄金十条”，实施更大力度的招商引资奖励办法，全面掀起新一轮招商引资热潮。开展“1·3·6·9·12”系列经贸活动，组织19场次推介活动，成功举办首届源商大会、首届中国·河源国际手机创新创业大赛。一批大型央企、民企落户，全年新签约项目240个，其中超亿元189个，超10亿元61个。

项目建设扎实推进。全年动工项目206个，投资总额955.4亿元；投产项目98个，投资总额155.1亿元。着力推进省、市重点项目建设，完成投资250亿元。强化重点项目用地保障，新增用地指标6290亩，清理批而未供、供而未用土地1.2万亩。中兴通讯河源基地完成25条生产线搬迁，深河国际模具产业城等项目完成年度建设任务，总投资100亿元的河源电厂二期、180亿元的阿里巴巴（河源）云计算数据中心等一批大项目动工建设。

（三）狠抓改革攻坚，发展活力不断增强。

以改革再出发的坚定态度，以提高社会生产力水平和优化营商环境为落脚点，全力推进供给侧结构性改革和重点领域改革。

供给侧结构性改革取得新进展。认真落实省“实体经济十条”，修订降低制造业企业成本政策措施，为企业减负超40亿元。完成化解钢铁行业过剩产能任务。银行机构杠杆率和国有企业资产负债率控制在合理水平。化解非商品房库存2.29万平方米。推进基础设施供给侧结构性改革，完成省重点项目外的补短板项目投资33.9亿元。完成交通基础设施投资108亿元，武深高速连平段建成通车，河惠莞高速建设加快推进，赣深高铁建设全面加快，国省道干线建设稳步推进，“四好农村路”建设全面铺开，新增通客运班车行政村101个。

关键领域改革扎实推进。启动“1+3+N”市级预算编制、执行和监督管理改革，零基预算改革和财政资金项目库改革全面铺开。新一轮市本级与源城区、市高新区财政事权和支出责任划分改革全面完成。国税地税征管体制改革顺利完成，环保税改革平稳落地，增值税、个人所得税改革稳步推进。出台市“金融十条”，率先完成农信社改制组建农商行任务。国企改革迈出关键步伐，出台深化市属国有企业改革实施方案。重点领域投融资机制改革有序推进。

营商环境持续优化。有序推进政府机构改革，稳步推进“放管服”改革，投资审批事项由123项压减至70项。成为全省“数字政府”改革政务云建设试点市，实现市、县、镇、村四级政务平台互联互通，率先推出“粤省事·河源通”并正式上线。授予江东新区和市高新区第二批市级管理权限128项。推进商事制度改革，企业开办时间压缩至4个工作日内，商事登记便利度全省排名第四。“三互”大通关改革扎实推进，综合保税区上报国务院审批，国际贸易“单一窗口”有效覆盖。完善公共资源交易信息化平台建设，实现四大类资源全流程电子化交易。

（四）狠抓创新驱动，发展动能不断释放。

大力实施创新驱动发展战略，积极谋划建设广深港澳科技创新走廊延伸区。

创新发展取得实效。着力培育创新主体，净增高新技术企业43家，全市存量达141家。加快创新平台建设，成立天津大学精密仪器院士工作站、深圳大学河源国际研究院，省科学院河源研究院建设顺利推进，河源广工大协同创新研究院工作成效明显；新增省企业重点实验室2家，实现省企业重点实验室零突破，新认定省级工程技术研究中心6家、省级新型研发机构2家。规上工业企业建立研发机构占比25.2%。开展产学研合作30项，带动相关产业新增产值5亿元以上。加强创新人才队伍建设，新引进中国工程院院士等高层次人才3人，新入选“珠江人才计划”创新科研团队1个。

创新环境不断优化。启动创新“七大行动计划”。专利申请量、专利授权量和发明专利授权量分别增长51.4%、28.7%和63.9%。落实科技创新政策，扶持科技企业资金7000多万元。促进科技金融融合发展，57家企业通过科技金融服务平台参与评级，授信总额度2.41亿元，19家企业获得贷款。

质量强市建设取得新成效。获国家级“标准科技创新”一等奖1项。“和平猕猴桃”成功申报国家地理标志保护产品。建成省新型墙体材料检验站、物联网产品检验站。产品质量抽查合格率93%以上，连续三年通过省质量工作考核。

（五）狠抓规划建设管理，城市品质不断提升。

突出规划建设管理一体化，着力谋划城市未来发展，为实施都市经济带动战略打好基础。

城市规划不断完善。高标准推进规划期至2035年的城市总体规划编制，完成城市发展战略、“十三五”近期建设规划编制，取得了城市轨道交通线网、新型城镇化和中心城区产业发展等专项规划成果。东江—新丰江水系连通工程和九潭水库项目纳入粤港澳大湾区专项发展规划，“三江六岸”生态城市功能廊道、高铁新城等规划编制有序开展。实现城乡规划建设管理队伍全覆盖。

城市建设扎实推进。大力推进“百街千巷”综合整治工程，改造提升市区背街小巷452条，改造市区水漫点47个。扎实推进“西优”工程，完成西环路旅游大道南段升级改造、福新北路（东源与源城间）贯通。客家乡土植物园建成开馆。黄子洞公园建成开放。新一路贯通、宝源大桥加宽、宝源新江片区新丰江两岸截污工程、东江公园二期等项目建设加快。实施市区供水管网改造工程，市区水源工程一期投入使用。完成市区100个老旧小区光纤入户改造。

城市管理力度加大。以“创文”、提升“国园”和“巩卫”为抓手，强化环境卫生、交通秩序、农贸市场和社区服务等综合整治，完成市政、园林、亮化等110多个整治建设项目，整治老旧小区9个，有效解决城市“六乱”等热点问题，“门前三包”责任制和城乡卫生日常保洁机制有效落实，城市管理水平不断提升。

（六）狠抓乡村振兴，农民人均收入首超全国平均水平。

坚持农业农村优先发展，大力实施乡村振兴战略，预计农民人均可支配收入14620元，增长9.9%，超过全国农民人均收入水平。

脱贫攻坚战取得实质成效。新增1.47万相对贫困人口达到脱贫标准。投入扶贫资金28.3亿元，新增扶贫项目4.5万个。大力实施产业扶贫，落实项目79648个，255个贫困村全面建立合作社，成功举办首届精准扶贫成果展示会。开展精准扶贫“回头看”，新纳入贫困人口2419人。深圳大力帮扶214个省定贫困村，投入资金16.8亿元，完成农业产业项目1775个。

美丽宜居乡村建设蓬勃开展。全域推进农村人居环境综合整治，完成省定贫困村“三清三拆三整治”任务。投入30.8亿元推进社会主义新农村示范村建设，完成自然村道路硬化1629公里，建成垃圾收集点6324个、卫生站184个，农村生活垃圾有效处理率96.6%，无害化处理率85%。解决99万农村人口饮水问题，完成中小河流治理179公里，完成农村危房改造2978户。建成移民美丽家园示范点10个，移民工作走在全省前列。

农村重点领域改革深入推进。农村土地确权登记颁证基本完成。农村集体产权制度改革稳步推进，22288个集体经济组织完成资产查漏补缺。建成农村产权流转管理服务县级平台7个，镇（街）级平台101个。政策性农业保险扩面提效，实现水稻种植保险全覆盖。普惠金融“村村通”工程提质增效，实现信用村和乡村金融服务站建设行政村全覆盖。

农业供给侧结构性改革不断发力。新增4个省级现代农业产业园、5个省级农业标准化示范区，建成农产品种植养殖企业质量安全示范点65个，新增省名牌产品12个。灯塔盆地核心区建设稳步推进，田园综合体国家试点建设加快，核心区干线公路全线建成通车，完成农产品加工孵化中心和综合服务中心大楼建设。确立农产品区域公用品牌——万绿河源，举办万绿河源·生态优品之十大高端农产品评选和庆祝首届中国农民丰收节活动。龙川国家级电子商务进农村综合示范县建设通过国家验收，东源县船塘镇（板栗）、紫金县龙窝镇（茶叶）入选全国农业产业强镇示范建设。完成粮食播种面积245万亩，连续多年完成省下达的粮食考评任务。

（七）狠抓环境治理和生态保护，生态环境质量居全省前列。

实现地表水考核断面水质优良率100%，县以上集中饮用水源水质达标率100%，水环境质量综合指数全省排名第一，环境空气质量优良率95.6%，全省排名第二。

大力推进环境治理。不折不扣推进中央环保督察“回头看”问题整改和省环保督察整改，关停、取缔、整改企业209家。认真实施新丰江水库水质保护三年行动计划，新丰江水库水质保持国家地表水Ⅰ类。境内东江干流水质保持地表水Ⅱ类。实现市、县、镇、村河长全覆盖，河长制工作全省排名前列。乡镇污水处理设施建设新开工22座，累计建成36座。开展大气环境综合整治专项行动和秋冬季大气污染防治攻坚战。全面启动土壤污染状况详查。被省列为地质灾害防治示范市，大力治理89个地质灾害隐患点。

深入推进生态建设。绿化河源大行动取得新成效，完成森林碳汇重点生态工程造林12.2万亩、生态景观林带3800亩，完成中央森林抚育及省级碳汇林抚育69.1万亩，建设乡村绿化美化示范点113个，新建湿地公园1个，新增省级生态公益林示范区1个，森林覆盖率达72.78%。实施林火远程视频监控系统二期建设，在全省率先实行森林防火工作挂牌跟踪制度，森林火灾受害率0.135‰，远低于省控1‰指标。推动省在东江流域率先启动生态补偿试点政策制定工作。建立市政府环境与发展综合决策咨询制度，科学划定生态红线，完成节能减排任务。积极开展全国水生态文明城市建设。

（八）狠抓民生实事，民生事业全面发展。

全年民生投入257.2亿元，占一般公共预算支出的76.8%。认真办好十件民生实事。

公共服务稳步提升。教育“创现”进程加速，源城、龙川创建成功，其余各县完成“创现”申报。新建4所、改扩建12所市区和县城中小学校，新增义务教育学位1.2万个。新改扩建54所农村义务教育寄宿制学校，增加寄宿制学位7070个。深化“强师工程”，推进“县管校聘”教师管理体制和农村小学师资培养机制改革。广东技术师范大学河源校区筹建工作加快推进。河职院成为粤东西北唯一通过验收的广东省示范性高等职业院校。省卫生强市建设加快，医药卫生体制改革有序推进，市公共卫生服务中心、市妇幼保健院二期建成使用，市中医院二期完成主体工程，深河人民医院加快建设。3个镇中心卫生院升级为县第二人民医院，完成36个乡镇卫生院标准化建设和334间村卫生站公建规范化建设。文体惠民提档升级，市图书馆、市文化馆、市档案馆被评为国家一级馆，市博物馆被评为国家二级馆。实现村级综合性文化服务中心全覆盖。开展各类公益性文化惠民活动1000余场，完成东源县阮啸仙故居等7处红色革命遗址修复提升工作。新建体育公园11个。成功举办“国际博物馆日”广东主会场宣传活动、萧殷文学研讨会暨萧殷文学馆开馆活动、南粤古驿道定向大赛、2018年河源万绿湖国际马拉松等群众文体赛事活动。

社会保障更加健全。全力推进扩面征缴，加快推进“互联网+社保”。深化企业职工养老保险和城镇职工医疗保险制度改革，优化大病保险政策，全面实行按病种分值付费。城乡低保、农村五保供养、孤儿保障标准大幅提高。完成退役军人信息采集，双拥优抚安置政策全面落实。社会福利慈善事业加快发展。推进人力资源服务产业园建设。城镇新增就业4.58万人，城镇登记失业率控制在2.3%以内。新增安置住房270套，新增分配公租房712套。

社会大局保持稳定。深化“平安河源”建设，实施“智慧新警务”战略，大力推进扫黑除恶专项斗争，深入开展“飓风2018”专项行动，打掉涉黑组织3个、恶势力犯罪集团13个、涉恶团伙126个。严格落实维稳责任制，实现“六个不发生”目标。开展打击走私“国门利剑2018”专项行动。深化全民禁毒工程，毒品违法犯罪有效遏制。推进“五星”平安村（居）建设和“一村（居）一法律顾问”“一村（居）一辅警”工作。成功打造“谁执法谁普法”责任制落实示范市和“智慧法援”先行地。强化重点领域安全隐患专项整治，安全生产形势总体稳定。食品药品安全形势稳中向好。

（九）狠抓党建工作，政府自身建设有力加强。

落实新时代党的建设总要求，坚持和加强党的全面领导，努力把政府系统各级党组织锻造得更加坚强有力。

旗帜鲜明讲政治。坚持以习近平新时代中国特色社会主义思想统领政府一切工作，全面贯彻落实党的十九大精神，深入学习贯彻习近平总书记重要讲话精神，树牢“四个意识”，坚定“四个自信”，坚决做到“两个维护”，抓好中央、省委巡视反馈意见整改，认真开展“两学一做”学习教育、“不忘初心、牢记使命”主题教育、“大学习、深调研、真落实”活动和专题民主生活会，强化市政府领导班子成员党建工作责任。

强化责任抓落实。坚持重大事项向市委请示报告，认真落实市人大及其常委会各项决定，主动接受市政协民主监督，办理人大代表建议120件、政协提案162件，办复率和满意率均为100%。严格落实“三重一大”集体决策制度，实行市政府常务会议题联审制度。出台《河源市人民政府“三定一督一通报”工作办法》，推动重点工作重点任务落到实处。

严明纪律建规章。严格落实中央八项规定，狠抓“四风”整治，抓好党风廉政建设，不断强化审计监督。加强法治政府建设，颁布首部实体规章《河源市城市市容和环境卫生管理办法》，落实行政首长出庭应诉制度，建立市政府立法咨询专家库，聘任首批30位立法咨询专家。

与此同时，统计、民族、宗教、外事、侨务、港澳、气象、老龄、殡葬、残疾人、妇女儿童、国防动员等工作取得新成绩。

各位代表！2018年的成绩来之不易。这是坚持用习近平新时代中国特色社会主义思想统领一切工作的结果，是省委、省政府和市委正确领导的结果，是市人大、市政协监督支持的结果，是全市人民共同奋斗的结果，是深圳、广州等兄弟市和海内外各界人士大力支持的结果。在此，我代表市人民政府，向全市广大干部群众，向驻河源部队、消防指战员和各民主党派、人民团体以及社会各界人士致以崇高的敬意！向长期关心支持我市改革发展的港澳同胞、台湾同胞、海外侨胞表示衷心的感谢！

各位代表！我们也清醒认识到，政府工作仍然面临严峻挑战。一是促进转型升级、建设现代产业体系任重道远。整体产业层次偏低，工业规模小，质量效益有待提高，传统产业改造升级任务艰巨；实体经济发展困难不少，用工难、融资难问题突出；新兴产业基础差，现有劳动力规模结构和素质能力无法满足现代产业发展需要。二是深化改革开放、加快创新发展需要攻坚克难。重点领域和关键环节改革有待加快，缺乏有效融入粤港澳大湾区建设的体制和平台，营商环境仍需优化；科技支撑和引领发展的能力仍需加大，企业创新主体作用发挥不够，新旧动能转换速度有待加快。三是保护生态环境、巩固核心优势任务仍然繁重。产业生态化绿色化水平不高，生态环境质量改善的成效还不稳固、持续向好的基础还不牢固，生态文明建设任务依然繁重。四是保障改善民生、加强社会治理力度有待加大。就业、教育、医疗、居住、养老等方面工作有待进一步加强，农村社会治理问题较为突出。五是转变政府职能、提高行政效能仍需下大功夫。政府部门服务质量和效率有待进一步提高，特别是干部的思想有待加快解放，作风有待改进，干事创业的精气神有待进一步提振，破解难题的担当作为有待进一步强化。对此，我们一定敢于直面问题，坚持问题导向和结果导向，勇于攻坚克难，积极推动经济社会实现高水平保护下的高质量发展。

二、2019年重点工作安排

2019年是新中国成立70周年，是全面建成小康社会关键之年，也是河源振兴发展提效破局之年，做好今年各项工作意义重大。今年政府工作的指导思想是：高举习近平新时代中国特色社会主义思想伟大旗帜，全面贯彻党的十九大和十九届二中、三中全会以及中央经济工作会议精神，深入贯彻习近平总书记重要讲话精神，认真落实省委十二届六次全会和市委七届六次、七次全会的决策部署，统筹推进“五位一体”总体布局，协调推进“四个全面”战略布局，围绕广东实现“四个走在全国前列”、当好“两个重要窗口”，坚持稳中求进工作总基调，坚持新发展理念，坚持推动高质量发展，坚持以供给侧结构性改革为主线，坚持深化市场化改革、扩大高水平开放，抢抓“四大机遇”，打好三大攻坚战，统筹做好稳增长、促改革、调结构、惠民生、防风险工作，进一步稳就业、稳金融、稳外贸、稳外资、稳投资、稳预期，深入落实“1+1+9”工作部署，全域全面融入粤港澳大湾区建设，加快建设现代化经济体系，大力建设人民幸福“心城”，奋力推动“示范区”“排头兵”和“两个河源”建设提效破局，为全面建成小康社会收官打下决定性基础，以优异成绩庆祝中华人民共和国成立70周年。

今年经济社会发展的主要预期目标是：地区生产总值增长7%左右，规模以上工业增加值增长7.5%，固定资产投资增长15%，进出口总额增长3%，社会消费品零售总额增长9%，地方一般公共预算收入增长6%，居民消费价格涨幅控制在2.8%以内，城镇登记失业率控制在3%以内，城乡居民收入增长与经济增长基本同步。

为实现各项目标任务，重点抓好九方面工作：

（一）主动融入粤港澳大湾区建设，努力构建全面开放新格局。

全面对接粤港澳大湾区专项规划，加大重点项目对接力度，全力打造国际科技创新中心重要拓展区、美丽湾区和宜居宜业宜游优质生活圈、世界级城市群的有机组成部分。

再掀交通建设热潮。以融入粤港澳大湾区交通一体化为主方向，高起点启动立体交通综合规划，启动高铁新城的交通枢纽组织建设。完成交通设施投资120亿元以上，确保河惠莞高速全线加快施工，其中龙川至紫金段建成通车；力争粤赣高速改扩建动工建设；加快龙寻、河惠汕等高速项目前期工作，提前谋划东西走向的河梅韶高速建设。加快赣深高铁建设，推动龙梅龙客专开工建设，谋划龙韶、龙汕等铁路建设。推进通用机场规划建设，推动东江航道河源段恢复通航，谋划广河客专、深惠城轨延伸至河源等重大铁路项目。力推国道G355紫金林田至高望段一级公路控制性工程开工建设。

全力创建省级全域旅游示范市。围绕建设粤港澳大湾区优质生活圈，打响“万绿河源”旅游品牌。做强旅游“拳头产品”，全力创建万绿湖国家5A级景区，推动巴伐利亚庄园建成国家级休闲度假区。加快东江百里画廊旅游公路、中央苏区留守红军纪念园、江东新区东江抗日战争革命遗址群等项目建设。实施乡村旅游“百镇千村”提质升级行动，加快乡村特色民宿建设。推进恐龙主题公园、休闲体育旅游特色小镇、水世界旅游和佗城国际旅游城、融创国际文旅城等项目招商和建设。

加快发展创新型经济。出台实施《河源市加快融入粤港澳大湾区建设 打造粤港澳大湾区国际科技创新中心重要拓展区行动计划》，主动接受广深港澳科技创新走廊的辐射带动。继续实施高新技术企业倍增和“树标提质”计划，力争高新技术企业突破160家。推进市高新区国家级知识产权示范区建设。推动符合条件的产业园区申报省级高新技术开发区。筹集科技信贷风险准备金，撬动科技型企业授信贷款1亿元以上。大力申报“珠江人才计划”“广东特支计划”“扬帆计划”，推动人才引进政策有效落地，加快人才公寓和专家楼建设。加快深圳大学河源国际研究院、广东技术师范大学河源研究院建设，推进天津大学精密仪器院士工作站提质增效。推动27%以上的规上工业企业建立研发机构。

持续掀起招商引资热潮。重点谋划2019年粤港澳大湾区“9+2”城市、北上杭等重点城市以及境外招商路演活动，加快构建全球招商网络，不断扩大河源城市影响力。深入落实重大招商项目市领导挂钩、督办考核制度，全力促进2018年“1·3·6·

9·12”系列经贸活动成果转化，推动项目加快落地动工建设。加快组建招商咨询委员会、投资顾问委员会和市投资咨询服务公司，整合扩充招商引资团队，大力培育主导产业专业招商协会，探索建立招商形象推广、项目准入布局、政策兑现落实、招商流程规范、产业要素激发高效衔接的全市招商引资“一盘棋”新格局，不断提升招商引资水平。

提升外经贸发展水平。大力推进综合保税区、保税物流中心（B型）建设，加快中欧班列、铁路口岸等平台建设，提升贸易便利化水平。充分利用进博会、广交会、广东海丝博览会等重点展会平台和香港贸发局平台，积极开拓“一带一路”新兴市场，培育跨境电商、市场采购等外贸新业态，引导企业巩固传统优势市场，助力企业扩大进出口空间。出台《促进电子商务提升发展三年行动计划》，大力创建国家、省电子商务示范县。

（二）坚持绿色发展，加快构建现代产业体系。

坚持产业生态化、生态产业化，推动产业转型升级和经济高质量发展，全力打造粤港澳大湾区现代产业重要战略腹地。

推动产业低碳绿色发展。深入落实供给侧结构性改革措施，加快淘汰落后产能和企业。推进钢铁和矿产等资源型传统制造业绿色化、现代化改造，培育壮大节能环保、清洁生产、清洁能源产业，推进资源全面节约和循环利用。推进工业技改转型升级，鼓励支持一批传统优势企业加快实施老旧设备更新与智能化改造，推动互联网、大数据、人工智能和制造业深度融合，工业技改投资增长10%。推动江东新区园区循环化改造，力争实现全市省级产业园循环化改造全覆盖。完成第二次全国污染源普查和年度节能减排任务。

推动工业高质量较快速度发展。按照“系统谋划、分类指导、重点突破”的思路进一步完善园区规划。大力实施园区提质增效五大行动计划：实施骨干企业提升行动计划，实施“一企一策”，降低企业运营成本，有重点扶持企业做大做强；实施园区承接主导产业转移行动计划，提升项目专业化承接转移水平，提升园区主导产业集聚效应，努力实现承接产业梯度转移项目50 个，电子信息产业产值达600亿元；实施产业合作共建行动计划，积极与深圳共建“飞地经济”，完成共建亿元以上项目 20个以上，引进落地10亿元以上项目8个以上，开工建设10亿元以上项目5个以上；实施投融资改革行动计划，发挥财政资金的撬动作用和放大效应，引进各类风险投资基金参与园区建设和主导产业发展，走市场化道路推动园区建设、招商、运营上新台阶；实施园区扩园提质行动计划，探索建立“亩均论英雄”工业园区考核评价机制。力争园区完成基础设施投入35亿元、“1·3·6·9·12”签约项目动工率和投产率分别达到70%和50%、园区规上工业增加值增长8.5%和全市新上规工业企业70家。推进军民融合产业发展。

大力发展现代服务业。推动生产性服务业向专业化发展和价值链高端延伸，大力发展总部经济、商贸物流、贸易结算、金融保险、风投创投等新业态，发展壮大都市经济。完善中心城区商业网点规划，力争新增上限商贸单位50家。大力实施技术标准和品牌带动战略，继续推进紫金春甜桔、东源仙湖茶国家地理标志保护产品申报工作，推动省质量监督车载智能网联产品检测站、果蔬产品检验站建设。完成无人机产品检验站建设。

发展壮大民营经济。坚持“两个毫不动摇”，全面落实省“民营经济十条”政策，出台我市的政策措施，着力解决民营经济发展面临的实际问题。鼓励民间资本进入市政公用、社会、文化事业等领域，参与战略性新兴产业、现代服务业和现代农业建设。创新政府配置资源方式，建立完善中小微企业信贷风险补偿、小额贷款保证保险、政策性融资担保等机制，设立中小微企业融资专项资金，为符合条件的企业提供贷款转贷服务，缓解企业“融资难、融资贵”问题。支持异地建立河源商会。建立民营企业投诉处理机制，积极稳妥解决民营经济历史遗留问题，构建“亲”“清”新型政商关系。

（三）狠抓项目建设，为高质量发展注入强劲动力。

谋划实施更多优质项目，全力推进135项省市重点项目建设，完成有效投资335亿元以上，掀起大抓项目的热潮。

健全高效推进项目建设机制。大力完善项目前期工作机制，构建项目发起、储备、启动和建设良性运行机制，统筹解决项目征地拆迁、建设资金等突出问题，加快推进阿里巴巴（河源）云数据中心、河源电厂二期等一批重点项目建设。大力抓好项目库建设，实行分类动态管理，用好用活中央苏区和革命老区等政策，力争更多项目纳入省的发展规划。

大力推进城建项目建设。落实好“十三五”近期建设规划。加快G205国道东移项目开工建设。谋划粤赣高速热水出入口建设，优化城北高速出入口及周边区域。按照轻重缓急和开发时序，用三年时间打通中心城区断头路，优先建设双下大桥和跨东江大桥。继续加快推进中山大道改造工程、建设大道下穿中山大道和文昌南路工程、红星东路贯通工程等项目建设；贯通内环路北段（西环路连接东环路）、新双下路（江东新区接老城区及宝源片区）等中心城区内环道路，解决交通“瓶颈”问题。

推进水利能源项目建设。加快实施新丰江坝下水环境综合整治。编制实施《河源市乡村振兴农村水利治理规划》，治理中小河流144公里。完成东源、和平1.12万亩高效节水灌溉工程建设，完成村村通自来水工程扫尾工作。积极谋划东江—新丰江水系连通和九潭水库建设项目。完成电网投资20亿元，确保220千伏连平江面输变电工程、联禾站主变扩建项目投产；加快800千伏“乌东德”直流接地极（连平段）工程和220千伏越王山输变电工程等项目建设。加快推动垃圾焚烧发电项目建设。

强化财税金融保障能力。落实减税降费措施，加快项目资金落地，统筹盘活财力，做大财政经济“蛋糕”。启动运作粤财河源投资产业发展引导基金，支持战略性新兴产业和科技创新型企业转型发展。推动市“金融十条”落地见效，实施股改5年财政扶持政策，推动企业上市挂牌。探索设立绿色发展专项资金以及发行绿色金融债券，鼓励支持绿色金融发展。加强政府性债务和隐性债务管理，完善金融防控机制，打好防控重大金融风险攻坚战，切实防范政府债务风险。

（四）大力实施乡村振兴战略，建设安居乐业的美丽家园。

集中力量掀起乡村振兴发展热潮，扎实推动乡村振兴出精品、造亮点，努力把“短板”变成“潜力板”。

坚决打好脱贫攻坚战。力争建档立卡贫困户全部达到预脱贫标准。突出抓好产业扶贫，实施“五个一”产业扶贫工程，促进产销对接，实现每个有劳动能力有参与意愿的相对贫困户都有扶贫产业带动。强化生态扶贫，让贫困群众在生态建设中积极参与、获得收益。坚持扶贫与扶志扶智相结合，推进就业扶贫，做好技能培训，提高贫困人口就业能力。强化财政资金攻坚保障，推进教育、住房、医疗、最低生活保障等扶贫政策全面落实。健全支持配合对口帮扶的工作机制，大力配合推进恒大集团扶贫项目。完善扶贫资金使用情况监督管理机制，深化扶贫领域腐败和作风问题专项治理。

推动农业高质量发展。围绕建设粤港澳大湾区的菜篮子、果盘子、米袋子、茶罐子，大力实施“一村一品、一镇一业”，探索建立农业大数据平台，利用云计算、人工智能等先进技术手段提高农产品的产量和销量，推动一二三产业融合发展。全力办好第二届广东省茶叶产业大会。加大农业标准化示范区创建和质量认证力度，实施农产品整体品牌形象“万绿河源”塑造工程。加快东源县国家农业可持续发展试验示范区建设。实现现代农业产业园县区全覆盖。促进综合服务体系建设。积极培育种养大户、家庭农场、农民专业合作社和农业龙头企业等经营主体，完善企业与农户的利益联结机制。完成93.23万亩粮食（水稻）生产功能区划定，粮食总产量稳定在88万吨以上。

建设生态宜居美丽乡村。坚持规划先行，基本实现村庄建设规划编制全覆盖。大力实施“百村示范、千村整治”工程，严格实施《河源市农村生活垃圾治理条例》，以打造省级新农村连片示范工程和省定贫困村创建社会主义新农村为突破口，全域整治提升农村人居环境。源城要高标准把五村连片开发打造成全市乡村振兴连片开发示范标兵；东源要把田园综合体建设成为全市乡村振兴综合开发排头兵；龙川、和平、连平和东源要全力支持打造粤赣省界原中央苏区（河源）、灯塔盆地示范区核心区2个乡村振兴示范带；紫金要全力抓好六镇连片示范创建。努力争创1-2个省级特色小镇，建成5-10个特色精品村。开展移民美丽家园建设。大力推进“厕所革命”。突出镇村联动，推动镇街建设提档升级。加快“四好农村路”建设，提高行政村客运通达率。

深化农村改革。深化农村土地管理制度改革，完善农村承包地“三权分置”的有效组织形式、经营方式和发展路径，探索推进农村宅基地“三权分置”，全域推进土地综合整治。加快高标准农田建设。制定引导农民互换并地的激励政策。用好城乡建设用地增减挂钩政策，统筹推进农村集体建设用地和宅基地拆旧复垦。深入推进农村集体产权制度改革，推动农村资源变资产、资金变股金、农民变股东改革。做好省扶持村级集体经济发展试点工作。加快普惠金融提质增效，提升农村金融服务能力。推动“放管服”改革向基层延伸，成立镇综合服务中心和村公共服务站，完善村级事务代办制度，提高乡村政务服务便利性。

（五）全面深化改革，打造一流营商环境。

以改革破解难题、激发活力，真正让市场在资源配置中起决定性作用，营造稳定公平透明、可预期的营商环境，厚植转型发展新优势。

深化“放管服”改革。稳妥有序完成政府机构改革，改革机构设置，优化职能配置，为各领域改革提供体制支撑和保障。深化镇街行政管理体制改革。加快商事制度改革，推广电子营业执照和全程电子化商事登记，推进“多证合一、一照一码”和“证照分离”改革。深入推进城建体系改革，理顺城建工作体系、城建分工体系、城建经营体系。深化行政审批制度改革，进一步压减权责清单事项。推进投资项目审批制度改革，优化审批流程，努力全面实现并联审批，力争审批时限压减一半。清理规范政府性基金和行政事业性收费，治理各种中介服务乱收费，降低企业运营成本。加快网上中介服务超市建设。深化市场监管综合改革，全面推行“双随机一公开”和“互联网+监管”，强化事中事后监管平台建设。完善商事纠纷仲裁机制。继续重视加强社会信用体系建设，实行守信联合激励和失信联合惩戒机制。

推进重点领域改革。深化市属国有企业改革，打造“5+2”具备自我造血功能的大企业或企业集团，加快推进市级经营性国有资产集中统一监管、国有“僵尸企业”出清重组、“三供一业”分离移交。深化市级预算编制执行监督管理改革，完善财政转移支付体制和基本公共服务领域事权划分，进一步理顺市、区财政管理体制。支持农村商业银行做大做强。

加快“数字政府”建设。大力推进政务信息化建设体制改革，构建“管运分离”的“数字政府”新体制。推动政务信息系统整合，破除“信息孤岛”，实现互联网和政务服务深度融合。积极开展政务服务“最多跑一次”和“三集中三到位”改革，完善“一站式服务”。推动“粤省事·河源通”高频民生服务事项上线，建成市级统筹、整体联动、部门协同、一网通办的“互联网+政务服务”体系，努力实现“让数据多跑路，群众少跑腿”。

（六）强化城市规划建设管理，打造现代化生态新城。

大力提升城市功能和品质，努力打造显山露水见绿、岭南客家风情浓郁的现代化生态新城。

高起点编制城市规划。做好新一轮城市总体规划编制；主动对接粤港澳大湾区规划建设，精心谋划“一主两副四组团”城市空间发展格局，强化中心城区核心带动作用，规划灯塔盆地和古竹—义容两个副中心。完善中心城区“1+6”空间规划体系。加强“三江六岸”生态城市功能廊道规划衔接，加快完成城市轨道交通线网规划，启动立体交通信息规划，完善各类控制性详细规划、专业规划，实现规划与项目有效对接。切实加强规划执行和土地储备工作。

进一步提升城市功能。统筹推进中心城区六大功能片区建设，加快“三江六岸”生态城市功能廊道建设，推进东江和新丰江市区段港口和码头规划建设，谋划实施东江西路中小企业总部经济带、江东新区城市起步区滨江功能带和老城区沿江文化提升带的规划建设。按照谋划总体概念规划、优化控制性详细规划、细化核心区修建性详细规划三个层次推进钓鱼台片区的规划建设，努力打造成河源的“城市客厅”。加快高铁新城建设，抓好站房周边交通组织、站前广场等基础设施建设，加快推进各类生产要素向高铁新城集聚。加快市民服务中心和不动产登记档案馆建设。加快推进粤港商务中心、国际会展中心、河源综合异地候机楼等重大城市功能体规划建设。

持续提升城市公共服务水平。深入推进“百街千巷”综合整治，重点加快管线和排水排污建设，完成278条普通街巷、30条示范街改造。结合“三旧改造”，打造文化街区。加快市区水源工程二期建设，完善城市供水体系，理顺管理体制，提高供水保障能力。抓好道路交叉口渠化改造。新建2个市区公共停车场，探索建设城市智能共享停车平台。完善公交站场建设，推行智慧公交（实时公交APP），提高公交服务水平。推进地下综合管廊与市政建设有机融合。强化海绵城市理念，加快建设迎客大道绿化工程、滨江大道公园等城市公园和城市水体，完善城市园林绿化景观配套。力促房地产市场平稳健康发展，新建1.2万套以上商品房。加快实施《河源市信息化基础设施三年行动计划（2018—2020年）》。

着力提升城市管理水平。深化城市管理领域综合执法改革，推进智慧城市建设，启动智慧城管二期项目，建设综合性城市管理数据库，推进城市管理基础信息互融共享，提高城市治理和服务智能化水平。提升国家园林城市管理水平，持续开展市容环境整治大行动。实施石峡垃圾场生态修复工程。加强城市防灾减灾管理，切实保障城市安全。

全力创建全国文明城市。深入开展社会主义核心价值观“1+X”工程，实施公民道德建设，发挥榜样模范和客家文化内涵的引领作用。打造河源志愿服务品牌。大力推进市、县、镇、村四级联创，深化文明村镇、文明单位、文明家庭等群众性精神文明创建活动。完成龙川县全省新时代文明实践中心试点建设，力争实现县区试点全覆盖。

（七）突出“三区并进”，推动都市经济与县域经济联动发展。

树牢全市经济一盘棋思想，加快发展要素流通，全力推动“三区”建设新突破和县域经济加快发展。

加快江东新区开发建设。科学谋划打造“三点三带四组团”，推动城市起步区、产业园区建设取得重大突破。启动江竹大道、省道S230线工程，扩大市区城市架构，加强与中心城区其它组团的互联互通。启动企业总部中心、金融服务中心、东江生态景观长廊等重大城市功能配套建设，发展大数据、新材料、高端装备制造和生命健康等主导产业，推进中国联通·河源云数据中心等项目建设，规划建设数据产业园、数据集聚区，培育发展数字经济。全面对接自贸区标准，推进“一枚公章管审批，一支队伍管执法”改革。积极争取省支持江东新区向国家申请设立县级行政区。

加快市高新区“二次创业”。发挥国家级高新区与省产业转移示范园区“双区”叠加的优势，加快与深圳南山建设“飞地经济”，深入开展“二次创业”，做大电子信息、精密机械制造等主导产业，培育扶持太阳能与光伏应用、新材料、食品饮料等特色优势产业。深化省科学院河源研究院、河源广工大协同创新研究院、深河金地创谷、深河创智产业园等重大创新平台建设，培育壮大科技创新型企业。加快建设中央活力区等重点区域，完善城市功能，创建全省绿色循环生态园区示范区。

加快灯塔盆地示范区先行发展。加快完善管理体制机制。强化科技支撑，大力创建国家农业高新技术产业示范区和省级综合性现代农业产业园。以建设粤港澳大湾区优质农副产品供应和集散基地为目标，高水平、高质量谋划和布局产业，建设农产品综合服务平台，探索实行农业企业代办制，培育和引进一批高新技术农业龙头企业，实施特色优势产业提质增效行动计划，大力发展生态设施与数字农业、食品加工业、农林生物与生命健康产业、绿色仓储与智能物流业。推进田园综合体示范试点项目建设，发展“农业+互联网”“农业+休闲观光旅游”新业态，打造一批农业专业镇。

加快提升县域经济实力。加快县城和中心镇新型城镇化建设，为发展县域经济提供强力支撑。注重分类指导，培育特色优势产业。源城区要充分发挥都市经济主战场主力军作用，大力发展以现代商贸旅游业为核心的现代服务业，以电子信息产业为核心的先进制造业，以精品农业为核心的现代农业。东源县要加快打造“1+8+1”产业发展新格局，大力发展电子信息、智能制造等主导产业，以富马为龙头加快新材料产业发展，推进盐东物流园建设。龙川县要积极构建“以建筑工业化为引领，电子电器、空气能、现代建筑多产业融合发展”的产业格局，推进总投资超过20亿元高端建筑材料生产基地等项目建设，努力打造国家级现代建筑工业化产业基地。和平县要发挥产城互动优势，努力扩园3.2平方公里，扎实推进智慧双创物流园建设。紫金县要坚持把紫城工业园作为工业发展的主战场，重点培育新电子、新材料、新能源产业，力争全年签约项目投资40亿元以上。连平县要以生态工业园升格为省级产业转移工业园为契机，加快“飞地经济”建设。

（八）守护一江清水和青山翠岭，厚植绿色优势。

深入践行习近平生态文明思想，全力保护好一江清水和青山翠岭，擦亮河源绿色生态品牌。

坚决打好污染防治攻坚战。实施打好污染防治攻坚战三年行动计划，巩固中央和省环保督察整改成效。打好碧水保优战，深入开展全国水生态文明城市建设，推进治水“三大行动”，强化东江、新丰江、枫树坝水库优良水体保护，建成新丰江水库环保项目50%以上，新丰江水库和东江干流水质分别保持地表水Ⅰ类和Ⅱ类标准。确保集中式饮用水源水质高标准达标，地表水考核断面优良水体比例达到100%，完成县级饮用水水源地环境问题清理整治，以东源县为试点实施千里碧道工程。打赢蓝天保卫战，实施全域联防联控，确保空气质量在全省排名前列。打好净土防御战，实施土壤环境分级分类管控，保障农产品质量和人居环境安全。加快市医疗废物处理中心迁建项目建设。实现乡镇污水处理设施全覆盖，完成50%的省定贫困村20户以上自然村污水设施建设。

构建现代环境治理制度体系。落实领导干部生态文明建设党政同责、一岗双责和终身追责制。全面落实河（湖）长制，建立健全东江流域突发环境事件应急联动机制。推动在东江流域开展跨地区生态补偿。开展环境产品交易试点，大力开发碳普惠制核证减排量项目，有序推进排污许可证制度改革。完善生态环境保护地方法规，强化环境监督执法，健全环境信用评价、资源环境承载能力监测预警机制。加快划定并严守“三条红线”，守护山水林田湖草生命共同体，提升生态系统质量。大力倡导绿色消费、绿色出行，推动形成简约适度、绿色低碳生活方式。

积极创建国家森林城市。全面启动创建国家森林城市工作。加强森林公园、湿地公园、城市公园、郊野公园和社区小公园等建设，启动迎客大道、滨江大道绿化工程，提升“三江六岸”绿化景观，着力“增色”“添花”。大力实施乡村绿化美化示范工程，继续推进新一轮绿化河源大行动，完成森林碳汇重点生态工程造林9万亩、森林抚育42.3万亩，建设绿美古树乡村5个，加快新丰江水库周边3.58万亩桉树林改造。

（九）着力增进民生福祉，打造人民幸福“心城”。

牢固树立以人民为中心的发展理念，努力增强人民群众的获得感、幸福感、安全感。

努力实现更高质量更充分就业。坚持就业优先战略和积极就业政策，统筹做好各类群体就业帮扶，促进女性平等就业。鼓励以创业带动就业，大力开展技能培训和创业培训，加大创业担保贷款支持力度。继续实施“客家菜师傅工程”。力争人力资源服务产业园建成使用，完善覆盖城乡的公共就业服务体系。新增城镇就业3万人，安置下岗失业人员再就业7000人、就业困难人员再就业1000人，促进创业1800人，带动就业5000人。

深入推进健康河源建设。推进市中医院、市妇幼保健院创建“三甲”专科医院，巩固市人民医院“三甲”成果。加强重点临床专科、卫生信息化建设，创建省级重点专科。加快深河人民医院、基层医疗卫生服务能力提升项目建设。推进公立医院药品跨区域联合集中采购改革，推进分级诊疗制度。加强与省内外各大医院优质专科合作，组建医联体或专科联盟，推动优质医疗资源更好服务群众。全面落实国家基本公共卫生服务项目，健全重大疾病及健康危害因素监测和防治体系，提升突发公共卫生事件应急处置能力。

加快教育现代化建设。完成教育“创现”工作任务。实施教育信息化2.0行动计划，促进信息技术与教育教学深度融合。启动基础教育提升三年行动计划。开展教育国际合作试点。加快广东技术师范大学河源校区建设。落实住宅小区配建中小学校（含幼儿园）政策，加大市区和县城中小学校以及公办幼儿园建设力度，增加学位供给。推进义务教育寄宿制学校建设，稳妥撤并农村“麻雀学校”。开展“流动学校”和“专递课堂”试点，加快补齐农村教育发展短板。推动对标新高考的普通高中课程改革。增强职业教育服务地方经济发展能力。提升特殊教育水平。深入实施“强师工程”，深化教师“县管校聘”管理体制改革，加快市、县（区）教师发展中心建设，启动优秀校长培养工程。推动市中小学综合实践基地建设。鼓励并规范社会力量办学。

推动文体事业协调发展。大力培育具有河源特色的客家文化、红色文化和绿色文化，夯实河源文化底蕴。实施文化惠民工程，继续推进村级综合性文化服务中心升级改造。推动市科技馆、市青少年宫、市妇女儿童活动中心建设，推进市美术馆建设和市图书馆、文化馆、博物馆、档案馆数字化建设。推进粤赣古驿道（河源段）的保护和开发，推动龙川至寻乌段粤赣古驿道开发建设。加快市非遗展馆建设，推进红色革命遗址修复，弘扬红色革命文化。大力开展“书香河源”全民阅读月等群众性文化活动。结合新中国成立70周年，组织创作一批文艺精品。推动举办河源国际恐龙文化资源保护开发研讨会。加快公共体育场地建设，广泛开展全民健身活动。加大体育人才培养和引进力度，拓宽竞技体育备战项目和渠道，积极申办国际皮划艇激流回旋赛。

加强社会保障体系建设。稳妥推进社保费和非税收入征管职责划转。积极推进参保扩面，实现城乡居民基本养老保险覆盖率99%以上，完善失业、工伤保险制度和大病保险制度，努力实现社会保险应保尽保。实施“互联网+社保”，扩大异地就医联网结算范围。落实重特大疾病医疗救助，完善最低生活保障和特困人员供养制度，进一步提高优待对象抚恤补助标准，加强残疾康复服务，扶持居家养老服务中心建设。做好困境儿童关爱保护。推进保障性安居工程建设。

强化社会治理创新。打好防范化解重大风险攻坚战，强化安全生产责任落实，坚决遏制较大以上事故发生；强化应急能力和管理体系建设，大力实施防灾减灾工程。深入开展扫黑除恶专项斗争、全民禁毒工作和突出违法犯罪专项打击行动。加大全民普法力度，全面推行“一村（居）一法律顾问”“一村（居）一辅警”和“雪亮工程”“智慧新警务”建设。落实打击生产销售假冒伪劣商品违法行为责任制和食品药品安全责任制。建成市食品药品检验技术中心。创建第十一届“全国双拥模范城”，努力实现“四连冠”。

办好十件民生实事。计划投入40亿元，其中市本级投入7.5亿元，全力办好年度十件民生实事。一是加快推进学前教育发展；二是提升基层医疗卫生服务能力；三是提高底线民生保障水平；四是加大力度促进就业创业；五是继续实施“百街千巷”综合整治工程；六是提升优化城区交通环境；七是丰富群众文体生活；八是提升便民利民服务水平；九是加大生态环境保护力度；十是提高市场食品质量安全水平。

加强统计、民族、宗教、外事、侨务、港澳、粮食、方志、气象、老龄、殡葬、社会组织、国防动员等工作。

各位代表！我们将始终牢记全心全意为人民服务的宗旨，努力建设人民满意的服务型政府。切实加强政府系统党的建设。全面贯彻习近平新时代中国特色社会主义思想和党的十九大精神，认真落实习近平总书记重要讲话精神，树牢“四个意识”，坚定“四个自信”，坚决做到“两个维护”，在思想上政治上行动上同以习近平同志为核心的党中央保持高度一致。推进“两学一做”学习教育常态化制度化，深入开展“不忘初心、牢记使命”主题教育和“大学习、深调研、真落实”活动。严明政治纪律和政治规矩，扎实抓好中央、省委巡视“回头看”反馈意见整改落实，努力营造风清气正的政治生态。着力推进法治政府建设。自觉接受市人大的法律监督和工作监督、市政协的民主监督和社会公众的舆论监督，广泛听取各民主党派、工商联、无党派人士和各人民团体的意见。坚持重大事项向市委、市人大报告，向市政协通报，认真办好人大代表议案建议和政协提案。认真做好政府规章和规范性文件报市人大备案审查工作。充分发挥政府在地方立法工作中的依托作用，为我市立法作出更多基础性贡献。大力推进政务公开，落实重大行政决策合法性审查和政府法律顾问制度，认真做好行政复议、行政应诉等工作。全力建设廉洁高效政府。切实履行管党治党主体责任和“一岗双责”，深入贯彻中央八项规定精神及实施细则，巩固“四风”整治成果，努力打造忠诚、干净、担当的高素质专业化干部队伍，全面提振广大干部干事创业精气神。加大对公共资金、公共资源交易等重点领域监管，强化审计监督。深入实施“三定一督一通报”工作办法，着力破解卡壳问题，确保中央和省以及市委的决策部署落地见效。

各位代表！让我们更加紧密地团结在以习近平同志为核心的党中央周围，高举习近平新时代中国特色社会主义思想伟大旗帜，凝心聚力、真抓实干，为把河源建设成为全省绿色发展的示范区、融入粤港澳大湾区的生态排头兵，走出生态河源、现代河源相得益彰的发展新路，为广东实现“四个走在全国前列”、当好“两个重要窗口”而努力奋斗！
